# Correlations between multiple obesity diagnostic criteria and fracture risk as well as cardiovascular diseases in patients with diabetes mellitus

**DOI:** 10.3389/fcvm.2026.1847739

**Published:** 2026-07-21

**Authors:** Zhenrun Zhan, Ke Zheng, Yalong Tu, Chao Lan, Lifeng Zheng

**Affiliations:** 1 Department of Endocrinology, The First Affiliated Hospital, Fujian Medical University, Fuzhou, China; 2 Department of Endocrinology, National Regional Medical Center, Binhai Campus of the First Affiliated Hospital, Fujian Medical University, Fuzhou, China; 3 Department of Orthopedics, National Regional Medical Center, Binhai Campus of the First Affiliated Hospital, Fujian Medical University, Fuzhou, China; 4 Department of Orthopedics, The First Affiliated Hospital of Fujian Medical University, Fuzhou, China

**Keywords:** cardiovascular diseases, diagnostic criteria, fracture risk, global burden of disease, obesity

## Abstract

**Objective:**

This study aimed to analyze the global disease burden of high body mass index (BMI) and to evaluate and compare the associations between three obesity diagnostic criteria—World Health Organization (WHO), European Association for the Study of Obesity (EASO), and American Obesity Association (AOU)—alongside the waist-to-height ratio (WHtR), with fracture risk and cardiovascular diseases (CVD) in a clinical population with diabetes mellitus.

**Methods:**

Data on the high BMI burden in China and globally (1990–2021) were sourced from the Global Health Data Exchange (GHDx). A cross-sectional clinical study enrolled 17,949 diabetic participants from a single center in China. Obesity was diagnosed using WHO, EASO, and AOU criteria. Logistic regression models were employed to analyze the associations between WHtR (analyzed as both a continuous variable and by tertiles) and CVD, high-risk hip fracture (HRHF), and high-risk major osteoporotic fracture (HRMOF), with adjustments for multiple covariates including demographic, lifestyle, and metabolic factors.

**Results:**

From 1990 to 2021, the age-standardized rate of high BMI in China increased by 146.22% (AAPC = 2.95%), exceeding the global increase of 70.89% (AAPC = 1.74%). In the clinical cohort, EASO criteria demonstrated significant associations with CVD. WHtR, as a continuous variable, was a significant risk factor for CVD and hip fracture (HF) across all models. When analyzed by tertiles, the highest WHtR tertile was associated with significantly increased risks of CVD (OR = 1.14, 95% CI: 1.04–1.25) and HF (OR = 1.83, 95% CI: 1.41–2.62) compared to the lowest tertile in fully adjusted models, showing a dose-response relationship. Restricted cubic spline models confirmed increases in risk with rising WHtR.

**Conclusion:**

The high BMI burden is rising markedly, especially in China. In this diabetic population, elevated WHtR was independently associated with higher risks of CVD and HF. Integrating WHtR into clinical practice could improve risk stratification for obesity-related comorbidities.

## 1 Introduction

Obesity is a multifactorial, complex, and chronic metabolic disorder with a rising global prevalence. According to the World Health Organization and the World Obesity Atlas 2025, approximately 2.548 billion adults worldwide were overweight or obese (BMI ≥ 25 kg/m^2^) in 2022 (1). This figure is projected to reach 2.9 billion by 2030, increasing the combined overweight and obesity rate to 50%. Notably, the population of adults with obesity (BMI ≥ 30 kg/m^2^) is expected to surge from 524 million in 2010 to 1.13 billion in 2030, representing an increase exceeding 115% (2). Obesity is closely linked to numerous chronic complications, including abnormal glucose metabolism (encompassing diabetes, pre-diabetes, and metabolic syndrome), cardiovascular diseases, liver disorders, dyslipidemia, gallbladder diseases, hypertension, osteoarthritis, and cancer.

Bone marrow-derived mesenchymal stem cells (BMMSCs) are pluripotent stem cells capable of differentiating into osteoblasts and possess multi-lineage differentiation potential (3). Adipogenic differentiation represents a key pathway that balances osteogenesis (4). Enhanced adipogenesis within BMMSCs contributes significantly to the pathogenesis of osteoporosis, as an increase in adipocytes leads to reduced bone mineral density, bone loss, and alterations in bone quality (5). Consequently, an intrinsic correlation exists between obesity and osteoporosis or osteoporotic fractures. Clarifying the role of adipogenesis will aid in elucidating the pathogenic mechanisms of osteoporosis. Research indicates that over half of postmenopausal women are affected by metabolic syndrome, with nearly 60% experiencing dyslipidemia (6). Disorders of lipid metabolism are frequently observed in patients with various typical forms of osteoporosis.

In contrast to general obesity assessed by BMI, anthropometric measures of central obesity are typically associated with elevated levels of health risk factors across diverse age groups and often serve as superior correlates (7–10). The boundary value of WHtR = 0.5 was initially proposed as a risk assessment tool two decades ago and has since demonstrated enhanced utility across multiple domains, including weight management and disease prediction (10–12). However, in cross-sectional analyses, these measures primarily reflect associations. This threshold has gained worldwide application, with evidence from numerous population studies reinforcing the premise that WHtR is a straightforward and effective anthropometric index, particularly for identifying health risks (13–16). Moreover, WHtR exhibits a more definitive association with mortality compared to BMI, beyond its established link with morbidity (17). Therefore, this study aimed to systematically examine the roles of obesity and lipid metabolism disorders in the pathogenesis and progression of cardiovascular diseases and osteoporosis. It further sought to investigate the correlation and associative efficacy of WHtR as a representative indicator for identifying the risk of cardiovascular diseases and osteoporotic fractures.

## 2 Materials and methods

### 2.1 Data sources of information on GBD

Data on the burden of high BMI (BMI ≥ 25 kg/m^2^) in China from 1990 to 2021 were obtained from the Global Health Data Exchange (GHDx), a repository developed as part of the GBD study initiated by the Institute for Health Metrics and Evaluation (IHME) at the University of Washington. The most recent dataset was released in 2021. Extracted data included all-age rates and age-standardized summary exposure rates attributable to high BMI (18, 19).

### 2.2 Statistical methods

Statistical analysis was performed using Joinpoint 4.9.0 and R 4.3.1 software. The average annual percentage change (AAPC) and its 95% CI were calculated using the Joinpoint regression model to analyze trends in the attributable disease burden of high BMI from 1990 to 2021. A *P*-value < 0.05 was considered statistically significant.

### 2.3 Clinical study

**Figure 1:**
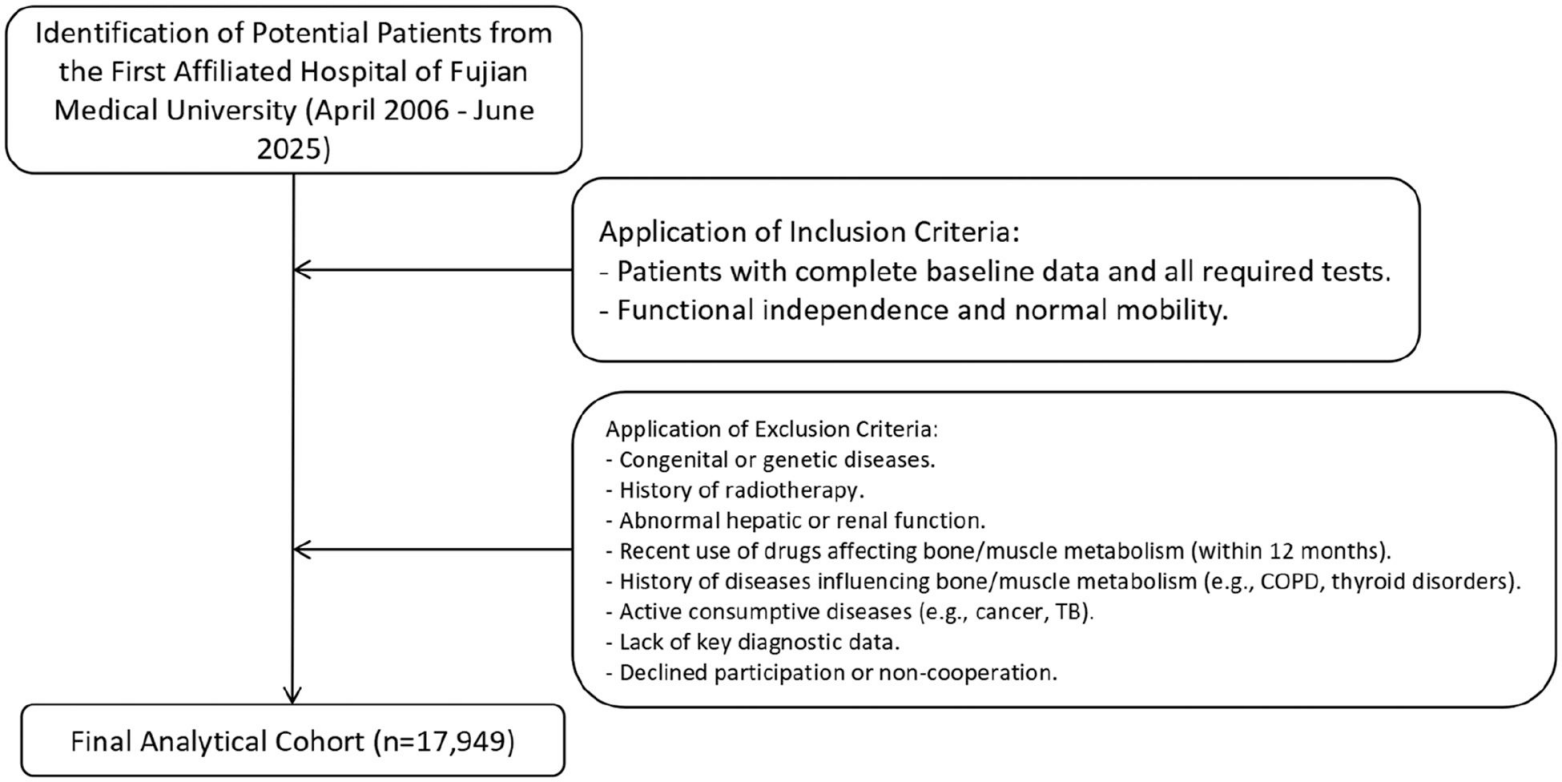
Flowchart of participant recruitment.

Study Population: This cross-sectional study enrolled 17,949 diabetic patients (9,561 males and 8,388 females; median age 63 years) from the Department of Endocrinology at the First Affiliated Hospital of Fujian Medical University between April 2006 and June 2025. Inclusion criteria were: (1) Patients examined or hospitalized at the study site during the specified period with complete baseline data; (2) Functional independence and normal mobility. Exclusion criteria were: (1) Congenital or genetic diseases; (2) History of radiotherapy; (3) Abnormal hepatic or renal function; (4) Recent (within 12 months) use of drugs affecting bone or muscle metabolism (e.g., corticosteroids, sex hormones, bisphosphonates); (5) History of diseases influencing bone or muscle metabolism (e.g., COPD, thyroid/parathyroid disorders, renal insufficiency, gastrointestinal diseases); (6) Active consumptive diseases (e.g., cancer, tuberculosis); (7) Lack of key diagnostic data; (8) Declined participation or non-cooperation (Figure 1). Data on demographics and clinical characteristics were collected from electronic medical records.

### 2.4 Research methodology

#### 2.4.1 Diagnosis of OP

1. According to the World Health Organization's (WHO) diagnostic criteria for osteoporosis (OP), bone mineral density (BMD) is measured using DXA scanning. The T-scores obtained categorize BMD into three levels: normal BMD (T-score ≥ −1.0 SD), osteopenia (T-score between −1.0 SD and −2.5 SD), and OP (T-score ≤ −2.5 SD) (20, 21).
2. Diagnostic criteria based on fragility fractures serve as another vital method for diagnosing osteoporosis: fractures that occur due to increased bone fragility when subjected to normal or mild external forces are also indicative of osteoporosis. Common sites include the vertebral bodies, hip, distal radius, proximal humerus, and pelvis (22).

#### 2.4.2 Diagnosis of obesity

Obesity was diagnosed according to the criteria established by three major working groups: the WHO (23), the EASO (24), and the AOU (25). Patients were categorized into four corresponding groups based on the diagnostic rules outlined in Table 1.

**Table 1:** Three diagnostic criteria for obesity (WHO, EASO, and AOU).

Parameter	Diagnostic criteria
WHO	BMI ≥ 30 kg/m^2^.
EASO	BMI ≥ 30 kg/m^2^ or BMI ≥ 25 kg/m^2^ with a waist-to-height ratio (WHtR) ≥ 0.5.
AOU	BMI ≥ 30 kg/m^2^ with at least one indicator of excess adiposity (waist circumference >88 cm in women or >102 cm in men, or elevated WHR or WHtR); or BMI < 30 kg/m^2^ with two or more adiposity indicators exceeding thresholds; while BMI ≥ 40 kg/m^2^ is automatically classified as obesity.

#### 2.4.3 Statistical analysis

Continuous data with normal distribution were presented as mean ± standard deviation, while non-normally distributed variables were described using median (interquartile range, IQR). Categorical variables were summarized as frequencies and percentages. Normality was assessed using the Kolmogorov–Smirnov test. Group comparisons were performed using the t-test or ANOVA for normally distributed data, and the Mann–Whitney U test or Kruskal–Wallis test for non-normal data. Binary logistic regression was employed to identify factors associated with OP, fracture risk, and CVD. Multivariable models incorporated clinically relevant covariates: Model 1 (unadjusted); Model 2 (adjusted for age, sex, and BMI); Model 3 [adjusted for age, sex, BMI, Hemoglobin, Red Blood Cell (RBC) Count, Triglycerides, High-Density Lipoprotein (HDL) Cholesterol, Neutrophil Count, and history of osteoporosis]. A three-knot restricted cubic spline regression model explored the relationship between WHtR and outcomes. WHtR was analyzed as a continuous variable or categorical variable (tertiles, with the first tertile as reference). Trend tests were conducted across tertile levels. Statistical significance was defined as a two-tailed *P* < 0.05. Analyses were performed using R 4.3.1 and SPSS 25.0.

## 3 Result

### 3.1 Trends in high BMI in China and globally

The age-standardized rate of high BMI in China increased from 7.754 (95% CI: 6.576–9.650) in 1990 to 19.092 (95% CI: 17.115–22.150) in 2021, reflecting a cumulative increase of 146.22%. Globally, the rate rose from 12.578 (95% CI: 11.139–14.745) to 21.494 (95% CI: 19.219–23.977), marking a cumulative increase of 70.89%. The AAPC for high BMI from 1990 to 2021 was 2.951% (95% CI: 2.937–2.964) in China, compared to 1.743% (95% CI: 1.735–1.752) globally (Table 2).

**Table 2:** All-age rates and age-standardized summary exposure rates for high BMI in China and globally (1990–2021).

Location	Measure	1990		2021		1990–2021 AAPC
		All-ages rates	Age-standardized rates per 100,000 people	All-ages rates	Age-standardized rates per 100,000 people	
		*n* (95% CI)	*n* (95% CI)	*n* (95% CI)	*n* (95% CI)	*n* (95% CI)
China	Male	7.819 (6.646–9.624)	7.966 (6.76–9.852)	19.748 (17.569–23.471)	19.486 (17.644–22.505)	2.9349 (2.9150–2.9549)
	Female	7.216 (6.064–9.089)	7.509 (6.325–9.457)	20.537 (17.676–24.54)	18.518 (16.439–21.609)	2.9579 (2.9358–2.9799)
	Both	7.527 (6.372–9.333)	7.754 (6.576–9.65)	20.134 (17.591–23.99)	19.092 (17.115–22.15)	2.9508 (2.9373–2.9643)
Global	Male	11.049 (9.838–12.946)	11.648 (10.252–13.776)	20.455 (18.167–23.085)	20.272 (18.083–22.787)	1.8030 (1.7918–1.8141)
	Female	12.699 (11.342–14.719)	13.439 (11.916–15.662)	23.255 (20.687–25.928)	22.666 (20.311–25.145)	1.6993 (1.6922–1.7065)
	Both	11.869 (10.585–13.841)	12.578 (11.139–14.745)	21.851 (19.438–24.492)	21.494 (19.219–23.977)	1.7434 (1.7347–1.7520)

### 3.2 Burden of high BMI across age groups

**Figure 2:**
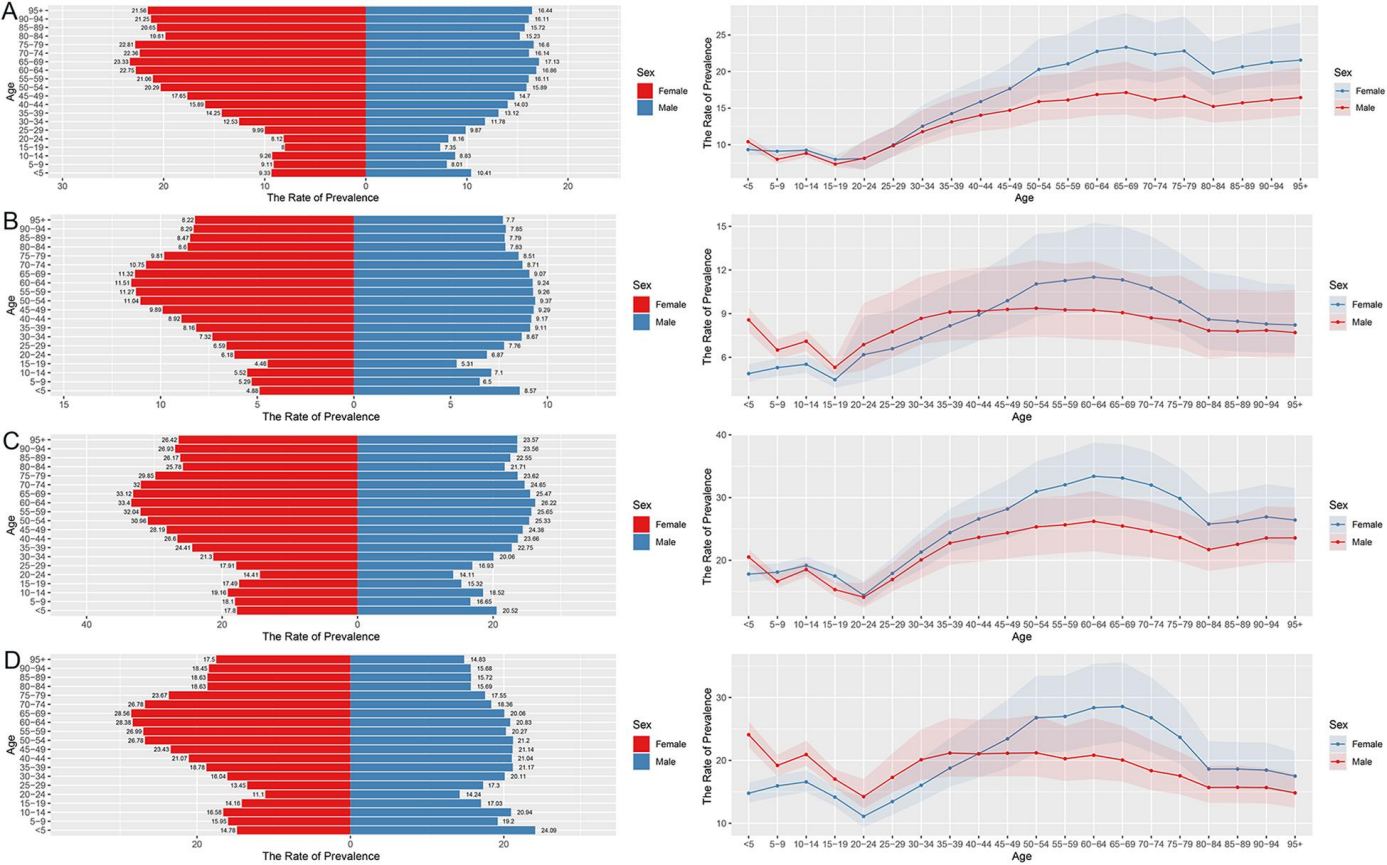
Summary exposure rates of high BMI across age groups. **(A)** Global 1990; **(B)** China 1990; **(C)** Global 2021; **(D)** China 2021.

Figure 2 illustrates the summary exposure rates of high BMI across different age groups in 1990 and 2021. Rates increased with advancing age, and the overall disease burden was significantly higher in 2021 than in 1990. Notably, the incidence peaked in the 60–69 age group. Interestingly, besides this persistent peak, another incidence peak emerged in the 10–14 age group.

### 3.3 Baseline characteristics

Preliminary logistic regression analysis revealed that EASO-defined obesity was associated with elevated risks of hip fracture and CVD, whereas other diagnostic criteria exhibited divergent association patterns (Supplementary Table S1). Consequently, our primary analysis prioritized the key metric underlying the EASO criteria, the Waist-to-Height Ratio (WHtR), as the principal exposure variable. The cohort (*n* = 17,949) had a median age of 63 years (IQR: 55–70) and a median WHtR of 0.54 (IQR: 0.50–0.58). Given the inconsistencies across different definitions and the physiological relevance of central adiposity, subsequent analyses focused on EASO-defined obesity and WHtR tertiles. Baseline characteristics stratified by EASO criteria are summarized in Supplementary Table S2. Furthermore, Table 3 presents the baseline characteristics stratified by WHtR tertiles, wherein participants were categorized into three groups according to WHtR levels: Q1 [0, 0.512], Q2 (0.512, 0.562], and Q3 (0.562, 1.307], with median WHtR values of 0.482, 0.536, and 0.596, respectively.

**Table 3:** Baseline characteristics stratified by WHtR tertiles.

variable	Total (*n* = 17,949)	Q1 (*n* = 5,941)	Q2 (*n* = 6,071)	Q3 (*n* = 5,937)	*p* value
osteoporosis, *n*(%)	3,066 (17.08)	984 (16.56)	1,023 (16.85)	1,059 (17.84)	0.15
Urea, mmol/L	5.49 (4.44,6.66)	5.40 (4.40,6.58)	5.53 (4.48,6.67)	5.54 (4.48,6.78)	<0.001
Uric acid, μmol/L	304.00 (244.00,373.10)	289.00 (228.00,353.60)	307.10 (247.90,375.40)	324.30 (258.30,391.00)	<0.001
Creatinine, μmol/L	63.00 (51.00,78.00)	62.00 (50.10,76.00)	64.00 (51.40,79.00)	62.70 (49.80,79.00)	<0.001
Systolic blood pressure, mm Hg	131.00 (119.00,145.00)	126.00 (115.00,139.00)	132.00 (120.00,145.00)	137.00 (124.00,150.00)	<0.001
Diastolic blood pressure, mm Hg	78.00 (70.00,85.00)	77.00 (69.00,84.00)	78.00 (71.00,85.00)	78.00 (71.00,86.00)	<0.001
Heart rate, beats/min	83.00 (74.00,91.00)	84.00 (75.00,93.00)	82.00 (74.00,91.00)	82.00 (74.00,90.00)	<0.001
FRAX fracture risk, %	3.10 (3.10,3.10)	3.10 (3.10,3.10)	3.10 (3.10,3.10)	3.10 (3.10,3.10)	<0.001
Hip fracture probability, %	1.80 (1.80,1.80)	1.80 (1.80,1.80)	1.80 (1.80,1.80)	1.80 (1.80,1.80)	<0.001
Cardiovascular diseases, *n*(%)	3,487 (19.43)	1,119 (18.84)	1,122 (18.48)	1,246 (20.99)	<0.001
Total cholesterol, mmol/L	4.41 (3.64,5.20)	4.40 (3.65,5.19)	4.41 (3.64,5.17)	4.43 (3.64,5.23)	0.34
Triglycerides, mmol/L	1.35 (0.93,1.95)	1.11 (0.79,1.66)	1.39 (0.98,1.97)	1.56 (1.10,2.24)	<0.001
HDL cholesterol, mmol/L	1.12 (0.93,1.37)	1.21 (1.00,1.50)	1.12 (0.92,1.33)	1.07 (0.90,1.27)	<0.001
LDL cholesterol, mmol/L	2.75 (2.06,3.47)	2.73 (2.06,3.45)	2.76 (2.06,3.48)	2.75 (2.05,3.49)	0.81
Neutrophil count, ×10^9^/L	3.73 (2.94,4.73)	3.56 (2.78,4.55)	3.75 (2.99,4.72)	3.90 (3.08,4.92)	<0.001
Monocyte count, ×10^9^/L	0.38 (0.30,0.45)	0.36 (0.28,0.42)	0.38 (0.30,0.44)	0.39 (0.31,0.47)	<0.001
Eosinophil count, ×10^9^/L	0.13 (0.08,0.18)	0.12 (0.07,0.17)	0.14 (0.08,0.19)	0.13 (0.09,0.19)	<0.001
Hemoglobin, g/L	133.00 (126.00,146.00)	134.00 (126.00,146.00)	134.00 (127.00,146.00)	131.00 (125.00,144.00)	<0.001
Lymphocyte count, ×10^9^/L	1.82 (1.45,2.25)	1.77 (1.40,2.17)	1.83 (1.46,2.27)	1.87 (1.49,2.31)	<0.001
WBC count, ×10^9^/L	6.35 (5.27,7.57)	6.07 (5.03,7.28)	6.38 (5.34,7.56)	6.59 (5.50,7.86)	<0.001
RBC count, ×10^12^/L	4.48 (4.12,4.87)	4.50 (4.12,4.89)	4.52 (4.17,4.90)	4.42 (4.07,4.81)	<0.001
Platelet count, ×10^9^/L	228.00 (188.00,270.00)	227.00 (187.00,269.00)	227.00 (188.00,270.00)	230.00 (191.00,271.00)	0.02
Albumin, g/L	42.50 (39.20,46.30)	42.60 (38.90,46.80)	43.00 (39.60,46.50)	42.00 (39.00,45.65)	<0.001
Alanine aminotransferase, U/L	20.00 (15.00,29.00)	19.00 (14.00,27.66)	21.00 (15.00,29.00)	22.00 (15.00,31.00)	<0.001
Aspartate aminotransferase, U/L	21.00 (17.00,25.13)	20.00 (16.00,25.13)	21.00 (17.00,25.13)	22.00 (17.00,25.13)	<0.001
Appendicular lean mass (ALM), kg	17.59 (14.72,20.80)	17.20 (14.30,20.19)	17.95 (14.80,21.03)	17.63 (15.04,21.27)	<0.001
ALM/BMI, (kg)/(kg/m^2^)	0.73 (0.62,0.85)	0.81 (0.68,0.92)	0.75 (0.63,0.85)	0.64 (0.56,0.77)	<0.001
Age, years	63.00 (55.00,70.00)	59.00 (51.00,66.00)	63.00 (56.00,70.00)	67.00 (59.00,73.00)	<0.001
Male, *n*(%)	9,561 (53.27)	3,541 (59.60)	3,459 (56.98)	2,561 (43.14)	<0.001
Height, cm	161.50 (155.00,168.00)	164.00 (157.00,169.50)	162.00 (156.00,168.00)	158.50 (153.00,165.00)	<0.001
Weight, kg	63.10 (55.90,71.00)	57.50 (51.00,64.10)	63.70 (57.00,70.20)	69.00 (61.60,77.00)	<0.001
BMI, kg/m^2^	24.13 (22.10,26.41)	21.60 (20.10,23.00)	24.19 (22.90,25.50)	27.24 (25.44,29.34)	<0.001
Waist circumference, cm	87.00 (80.00,93.00)	78.00 (74.00,82.00)	87.00 (83.50,90.00)	96.00 (91.00,100.00)	<0.001
Hip circumference, cm	93.00 (89.00,98.00)	89.00 (86.00,92.00)	93.00 (90.00,97.00)	99.00 (94.00,103.00)	<0.001
Waist to hip ratio	0.93 (0.88,0.97)	0.88 (0.84,0.91)	0.93 (0.90,0.96)	0.97 (0.94,1.01)	<0.001
AOU obesity, *n*(%)	12,435(69.28)	993(16.71)	5,539(91.24)	5,903(99.43)	<0.001
WHO obesity, *n*(%)	1,225(6.82)	10(0.17)	44(0.72)	1,171(19.72)	<0.001

### 3.4 Primary outcome

When analyzed as a continuous variable, WHtR × 10 was significantly associated with an increased risk of CVD across all models (Model 1: OR = 1.13, 95% CI: 1.06–1.19, *P* < 0.001; Model 2: OR = 1.14, 95% CI: 1.07–1.21, *P* < 0.001; Model 3: OR = 1.17, 95% CI: 1.09–1.23, *P* < 0.001) and HRHF (Model 1: OR = 1.63, 95% CI: 1.42–1.89, *P* < 0.001; Model 2: OR = 1.37, 95% CI: 1.16–1.61, *P* < 0.001; Model 3: OR = 1.44, 95% CI: 1.21–1.65, *P* < 0.001). In contrast, the association with HRMOF was not statistically significant in any model; the continuous variable showed a marginal positive trend in Model 3 (OR = 1.53, 95% CI: 1.04–2.13, *P* = 0.091) but did not reach conventional significance.

**Figure 3:**
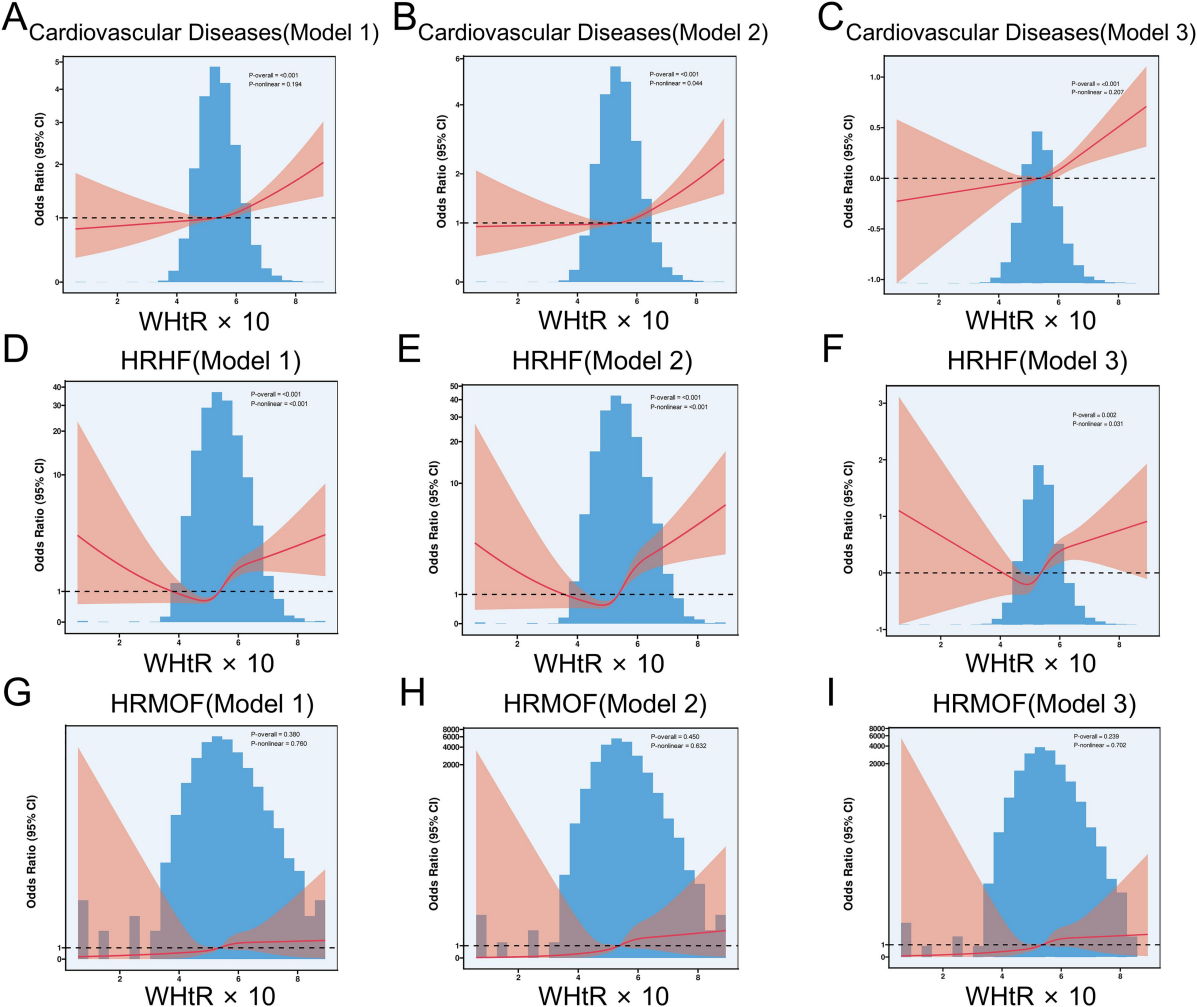
Restricted cubic spline curves for WHtR hazard ratios. **(A–C)** CVD; **(D–F)** HRHF; **(G–I)** HRMOF.

When analyzed by tertiles, compared to Q1 (reference), the highest tertile (Q3) was associated with a significantly increased risk of CVD in Model 3 (OR = 1.18, 95% CI: 1.06–1.28, *P* = 0.019) and HRHF in Model 3 (OR = 1.83, 95% CI: 1.41–2.62, *P* < 0.001). For HRMOF, no significant association was observed with Q3 in any model (Model 3: OR = 2.13, 95% CI: 0.84–6.64, *P* = 0.141). Trend tests revealed a significant dose-response relationship for CVD (*P* for trend = 0.012) and HRHF (*P* for trend <0.001), but not for HRMOF (*P* for trend = 0.132) in the fully adjusted model (Table 4, Figure 3).

**Table 4:** Logistic regression odds ratios (ORs) for CVD and fracture risk.

Categories	Model 1		Model 2		Model 3	
	OR (95%CI)	*P*-value	OR (95%CI)	*P*-value	OR (95% CI)	*P*-value
Cardiovascular Diseases						
Continuous variable	1.13 (1.06, 1.19)	<0.001	1.14 (1.07, 1.21)	<0.001	1.17 (1.09,1.23)	<0.001
Tertile						
Q1 (*n* = 5,941)	ref		ref		ref	
Q2 (*n* = 6,071)	0.98 (0.90, 1.08)	0.725	0.97 (0.88, 1.06)	0.486	0.91 (0.84,1.02)	0.352
Q3 (*n* = 5,937)	1.14 (1.04, 1.25)	0.004	1.14 (1.04, 1.25)	0.007	1.18 (1.06,1.28)	0.019
*P* for trend		0.003		0.005		0.012
HRHF						
Continuous variable	1.63 (1.42,1.89)	<0.001	1.37 (1.16,1.61)	<0.001	1.44 (1.21,1.65)	<0.001
Tertile						
Q1 (*n* = 5,941)	ref		ref		ref	
Q2 (*n* = 6,071)	1.35 (0.96, 1.88)	0.081	1.11 (0.79, 1.56)	0.539	1.26 (0.87,1.82)	0.506
Q3 (*n* = 5,937)	2.53 (1.87, 3.41)	<0.001	1.75 (1.29, 2.40)	<0.001	1.83 (1.41,2.62)	<0.001
*P* for trend		<0.001		<0.001		<0.001
HRMOF						
Continuous variable	1.47 (0.96, 2.26)	0.078	1.56 (0.89, 2.72)	0.120	1.53 (1.04,2.13)	0.091
Tertile						
Q1 (*n* = 5,941)	ref		ref		ref	
Q2 (*n* = 6,071)	0.78 (0.29, 2.08)	0.614	0.88 (0.31, 2.55)	0.817	0.82 (0.41,2.23)	0.781
Q3 (*n* = 5,937)	1.89 (0.84, 4.24)	0.123	2.26 (0.71, 7.19)	0.168	2.13 (0.84,6.64)	0.141
*P* for trend		0.092		0.149		0.132

Model 1 (unadjusted); Model 2 (adjusted for age, sex, and BMI); Model 3 (adjusted for age, sex, BMI, Hemoglobin, RBC Count, Triglycerides, HDL Cholesterol, Neutrophil Count, and history of osteoporosis).

### 3.5 Subgroup analysis

**Figure 4:**
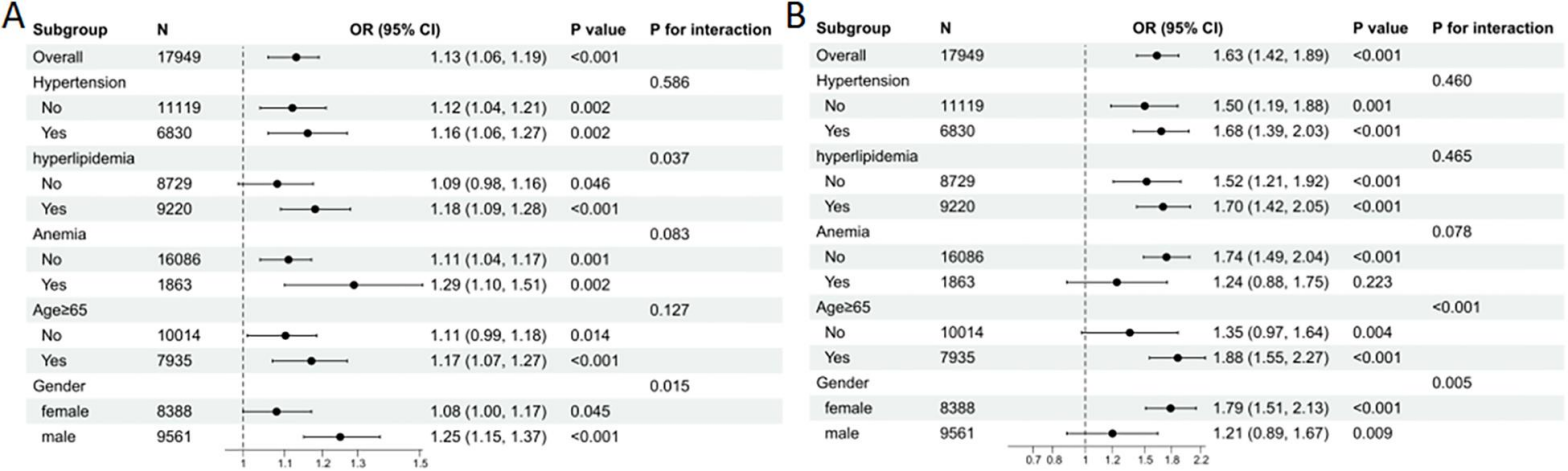
Forest plots of ORs for **(A)** CVD and **(B)** HRHF across subgroups.

Subgroup analyses demonstrated that elevated WHtR was consistently associated with increased risks of CVD and HRHF across all strata, including sex, age (<65 vs. ≥65 years), hypertension, hyperlipidemia, and anemia (Figure 4).

## 4 Discussion

Global Burden of Disease studies indicate that summary exposure values for high BMI continue to rise in China and worldwide, aligning with the increasingly severe global obesity epidemic. By 2022, over 1 billion people worldwide were affected by obesity (26). In this cross-sectional study, obesity diagnosed according to EASO criteria was not only significantly associated with CVD and OP but also showed clear correlations with high-risk hip fracture (HRHF).

Obesity is a major global public health challenge closely linked to cardiovascular risk factors, including dyslipidemia, hypertension, insulin resistance, and metabolic syndrome (27). Beyond metabolic abnormalities, obesity itself is an independent risk factor for CVD (28). Mechanistically, obesity triggers a state of inflammation by elevating pro-inflammatory mediators (e.g., IL-6, TNF-α) and reducing adiponectin, potentially accelerating atherosclerosis and oxidative stress, ultimately leading to major adverse cardiovascular events (29). Concurrently, obesity is a significant risk factor for OP and osteoporotic fractures.

The traditional view that fat accumulation protects bones is being challenged. Based on the balance between osteogenesis and adipogenesis, the expansion of bone marrow adipose tissue is common in high-risk OP populations, leading to reduced bone formation (30). Studies show a significant negative correlation between visceral adipose tissue and bone density (30). While obesity increases mechanical load, which may increase traumatic fracture risk, a population-based study indicated a positive correlation between the weight-adjusted waist circumference index and hip/spine fractures, suggesting that fat redistribution and muscle infiltration play critical roles in fracture pathogenesis (31).

The concept of metabolically healthy obesity (MHO), defined based on BMI (23), describes individuals who are overweight but have normal metabolic profiles, including normal insulin sensitivity and low inflammation (32). Research suggests that metabolic health status is a more important determinant of cardiovascular outcomes than obesity itself (33, 34). Therefore, relying solely on BMI for cardiovascular risk assessment remains controversial.

This study employed EASO, AOU, and WHO criteria alongside WHtR. Our results demonstrate that EASO criteria provide a more comprehensive assessment of fat distribution, showing stronger associations with CVD. EASO-defined obesity correlated with higher levels of metabolic and inflammatory parameters and a higher prevalence of CVD. Conversely, OP prevalence and HDL-C levels were lower in this group. Analysis by WHtR tertiles revealed a positive correlation with CVD risk and HRHF scores, persisting after adjustment.

A systematic review indicated that WHtR is superior to BMI and waist circumference in associating with CVD risk (13). As an effective indicator for assessing visceral adipose tissue (35), WHtR has been recommended to replace BMI for fat assessment (36). Recent NHANES data showed that incorporating WHtR significantly improves associative ability for CVD mortality (37). Evidence from the UK Biobank shows a linear association between WHtR and ischemic CVD, myocardial infarction, and stroke (38). Furthermore, WHtR correlates positively with coronary artery calcification, suggesting its potential as a biomarker for subclinical atherosclerosis (39). Central obesity induces adipose tissue dysfunction and chronic systemic inflammation, forming the basis for cardiovascular complications (40). Our findings confirm that EASO criteria, which incorporate WHtR, perform better in identifying CVD than AOU or WHO criteria.

The core value of this study lies in breaking through the limitations of relying solely on BMI assessment. For the first time, it systematically demonstrates the dual associative value of integrating WHtR into the EASO diagnostic framework for CVD and osteoporotic fractures within a diabetic population. The fundamental reason EASO criteria outperform WHO standards is that BMI cannot distinguish heterogeneity in fat distribution (41–43). In contrast, EASO accurately identifies visceral fat accumulation—a critical pathophysiological link—by incorporating WHtR (44). Notably, while AOU criteria include multiple indicators, they did not demonstrate superior clinical net benefit compared to EASO in this cohort. This suggests that the simple yet efficient WHtR metric may be a more cost-effective tool for risk stratification (45, 46). This difference is not coincidental; mechanistically, central obesity (high WHtR) is accompanied by adipose tissue dysfunction, a cytokine storm, and lipotoxicity, which directly drive endothelial injury and atherosclerosis (47–49).

More importantly, this study challenges the traditional “obesity-bone” paradigm and offers a fresh perspective on the “obesity paradox.” For decades, high BMI has been viewed as mechanically protective for bone, reflected in WHO criteria showing reduced OP risk among obese individuals (OR = 0.598) (50). However, our refined phenotypic analysis reveals that when obesity manifests central distribution (high WHtR, i.e., >0.562), it confers not protection but significantly increased fracture risk (highest tertile OR = 2.53) (51). This association extends beyond mechanical overload. While increased weight augments skeletal loading, bone loss in central obesity is primarily driven by alterations in the bone marrow microenvironment—specifically, the encroachment of adipocytes on bone-forming units, enhanced adipogenic differentiation leading to suppressed osteogenesis, and chronic systemic inflammation upregulating bone resorption (52, 53). Thus, central obesity masked by BMI mediates elevated fracture risk through inflammation and metabolic dysregulation.

## 5 Conclusion

The study reveals a concerning trend of continuously rising high BMI prevalence globally and in China. Elevated WHtR demonstrates significant cross-sectional associations with both CVD incidence and hip fracture risk. Regular monitoring and management of WHtR as a correlate for adverse clinical outcomes hold crucial clinical significance. Healthcare providers should specifically focus on individuals with WHtR levels exceeding 0.562 and implement proactive interventions to improve patient prognosis.

## 6 Limitation

This study has several limitations. First, the single-center cross-sectional design limits our ability to infer causality. While we observed strong associations, we cannot conclude that WHtR causes CVD or fractures. Prospective cohort studies are needed to confirm these findings. Second, although we utilized a large sample size, selection bias is inherent to single-center hospital-based studies, limiting generalizability. Third, a major limitation is the potential for incorporation bias, as WHtR is part of the EASO definition. To mitigate this, we focused our main interpretation on WHtR as a continuous variable, independent of the EASO classification. Fourth, despite adjusting for multiple covariates in Model 3, residual confounding by unmeasured factors (such as dietary habits, physical activity levels, and detailed medication histories) may still exist.

## Data Availability

The raw data supporting the conclusions of this article will be made available by the authors, without undue reservation.
