# Supplementary material for: Correlations between multiple obesity diagnostic criteria and fracture risk as well as cardiovascular diseases in patients with diabetes mellitus
Source: Front Cardiovasc Med. 2026 Jul 21;13:1847739. doi: 10.3389/fcvm.2026.1847739 (PMC13433434; doi:10.3389/fcvm.2026.1847739)
Supplement: Supplementary file 1 [file Table1.docx]

Supplementary Table 1. Logistic regression analysis of obesity defined by WHO, EASO, and AOU criteria.

| Diagnostic criteria | Osteoporosis OR (95% CI) | HRMOF OR (95% CI) | HRHF OR (95% CI) | CVD OR (95% CI) |
| --- | --- | --- | --- | --- |
| BMI Obesity (WHO) | 0.598(0.499-0.717)*** | 0.504(0.258-0.983)* | 0.780(0.470-1.295) | 1.063(0.920-1.228) |
| EASO Obesity | 0.675(0.621-0.733)*** | 1.248(0.971-1.604) | 1.637(1.300-2.061)*** | 1.250(1.159-1.347)*** |
| AOU Obesity | 1.043(0.959-1.136 | 3.186(2.184-4.646)*** | 1.751(1.316-2.329)*** | 1.076(0.992-1.166) |

Note: *: p < 0. 05; **: p < 0. 01; ***: p < 0. 001.

Supplementary Table 2. Baseline characteristics stratified by EASO criteria.

| **Parameter** | **Total (n=17949)** | **EASO non-obesity (n=10963)** | **EASO obesity (n=6986)** | **P value** |
| --- | --- | --- | --- | --- |
| **osteoporosis, n(%)** | 3066(17.08) | 2102(19.17) | 964(13.80) | <0.001 |
| **Urea, mmol/L** | 5.49(4.44,6.66) | 5.45(4.41,6.64) | 5.53(4.50,6.70) | 0.29 |
| **Uric Acid, μmol/L** | 304.00(244.00,373.10) | 293.60(233.10,357.20) | 330.90(266.00,396.28) | <0.001 |
| **Creatinine, μmol/L** | 63.00(51.00,78.00) | 61.60(49.40,76.00) | 65.00(52.00,80.00) | 0.28 |
| **Systolic Blood Pressure, mm Hg** | 131.00(119.00,145.00) | 129.00(117.00,142.00) | 135.00(123.50,148.00) | <0.001 |
| **Diastolic Blood Pressure, mm Hg** | 78.00(70.00,85.00) | 77.00(70.00,84.00) | 79.00(72.00,87.00) | <0.001 |
| **Heart Rate, beats/min** | 83.00(74.00,91.00) | 83.00(75.00,92.00) | 82.00(74.00,90.00) | 0.66 |
| **FRAX FractureRisk, %** | 3.10(3.10,3.10) | 3.10(3.10,3.10) | 3.10(3.10,3.10) | 0.41 |
| **Hip Fracture Probability, %** | 1.80(1.80,1.80) | 1.80(1.80,1.80) | 1.80(1.80,1.80) | <0.001 |
| **Cardiovascular Diseases, n(%)** | 3487(19.43) | 1979(18.05) | 1508(21.59) | <0.001 |
| **Total Cholesterol, mmol/L** | 4.41(3.64,5.20) | 4.41(3.64,5.19) | 4.42(3.64,5.20) | 0.66 |
| **Triglycerides, mmol/L** | 1.35(0.93,1.95) | 1.23(0.85,1.85) | 1.55(1.09,2.21) | <0.001 |
| **HDL Cholesterol, mmol/L** | 1.12(0.93,1.37) | 1.16(0.96,1.43) | 1.07(0.90,1.27) | <0.001 |
| **LDL Cholesterol, mmol/L** | 2.75(2.06,3.47) | 2.74(2.05,3.48) | 2.76(2.06,3.46) | 0.59 |
| **Neutrophil Count, ×10⁹/L** | 3.73(2.94,4.73) | 3.67(2.86,4.69) | 3.83(3.05,4.79) | <0.001 |
| **Monocyte Count, ×10⁹/L** | 0.38(0.30,0.45) | 0.37(0.29,0.44) | 0.39(0.31,0.46) | <0.001 |
| **Eosinophil Count, ×10⁹/L** | 0.13(0.08,0.18) | 0.13(0.07,0.18) | 0.14(0.09,0.19) | <0.001 |
| **Hemoglobin, g/L** | 133.00(126.00,146.00) | 132.00(125.00,144.00) | 134.00(128.00,147.00) | <0.001 |
| **Lymphocyte Count, ×10⁹/L** | 1.82(1.45,2.25) | 1.78(1.41,2.20) | 1.90(1.51,2.32) | <0.001 |
| **WBC Count, ×10⁹/L** | 6.35(5.27,7.57) | 6.23(5.16,7.45) | 6.55(5.48,7.76) | 0.67 |
| **RBC Count, ×10¹²/L** | 4.48(4.12,4.87) | 4.45(4.09,4.84) | 4.53(4.18,4.91) | <0.001 |
| **Platelet Count, ×10⁹/L** | 228.00(188.00,270.00) | 228.00(188.00,272.00) | 227.00(190.00,267.00) | 0.03 |
| **Albumin, g/L** | 42.50(39.20,46.30) | 42.40(38.90,46.40) | 42.70(39.60,46.30) | 0.08 |
| **Alanine Aminotransferase, U/L** | 20.00(15.00,29.00) | 19.00(14.00,27.66) | 23.00(16.00,33.00) | <0.001 |
| **Aspartate Aminotransferase, U/L** | 21.00(17.00,25.13) | 20.00(16.00,25.13) | 22.00(17.00,26.00) | <0.001 |
| **ALM, kg** | 17.59(14.72,20.80) | 16.53(13.99,19.61) | 19.48(16.18,22.66) | <0.001 |
| **ALM/BMI, (kg)/(kg/m²)** | 0.73(0.62,0.85) | 0.75(0.63,0.87) | 0.70(0.58,0.82) | <0.001 |
| **Age, years** | 63.00(55.00,70.00) | 62.00(54.00,70.00) | 64.00(56.00,72.00) | <0.001 |
| **Male, n(%)** | 9561(53.27) | 5844(53.31) | 3717(53.21) | 0.91 |
| **Height, cm** | 161.50(155.00,168.00) | 161.50(155.00,168.00) | 161.50(155.00,167.50) | 0.02 |
| **Weight, kg** | 63.10(55.90,71.00) | 58.10(52.30,64.00) | 71.70(65.70,78.20) | <0.001 |
| **BMI, kg/m²** | 24.13(22.10,26.41) | 22.60(21.00,23.80) | 27.18(25.91,29.01) | <0.001 |
| **Waist Circumference, cm** | 87.00(80.00,93.00) | 82.00(77.00,87.00) | 94.00(90.00,99.00) | <0.001 |
| **Hip Circumference, cm** | 93.00(89.00,98.00) | 90.00(87.00,94.00) | 99.00(95.00,103.00) | <0.001 |
| **Waist to Hip Ratio** | 0.93(0.88,0.97) | 0.91(0.87,0.95) | 0.96(0.92,0.99) | <0.001 |
| **Waist to Height Ratio** | 0.54(0.50,0.58) | 0.51(0.48,0.54) | 0.58(0.55,0.62) | <0.001 |
| **AOU Obesity, n(%)** | 12435(69.28) | 5826(53.14) | 6609(94.60) | <0.001 |
| **WHO Obesity, n(%)** | 1225( 6.82) | 0(0) | 1225(17.54) | <0.001 |
